# Lck Function and Modulation: Immune Cytotoxic Response and Tumor Treatment More Than a Simple Event

**DOI:** 10.3390/cancers16152630

**Published:** 2024-07-24

**Authors:** Juan Bautista De Sanctis, Jenny Valentina Garmendia, Hana Duchová, Viktor Valentini, Alex Puskasu, Agáta Kubíčková, Marián Hajdúch

**Affiliations:** 1 Institute of Molecular and Translational Medicine, Faculty of Medicine and Dentistry, Palacky University, 77900 Olomouc, Czech Republic; jenny.garmendia@gmail.com (J.V.G.); viktor.valentini@upol.cz (V.V.); agata.kubickova@upol.cz (A.K.); marian.hajduch@upol.cz (M.H.); 2 Czech Advanced Technologies and Research Institute (CATRIN), 77900 Olomouc, Czech Republic; 3 Faculty of Science, Palacky University, 77900 Olomouc, Czech Republic; hana.duchova01@upol.cz (H.D.); alex.puskas02@gmail.com (A.P.); 4 Laboratory of Experimental Medicine, University Hospital Olomouc, 77900 Olomouc, Czech Republic

**Keywords:** Src kinases, Lck, hematologic tumors, solid tumors, T-cell cytotoxicity, NK cytotoxicity, CAR-T cell, cell proliferation, cell adhesion

## Abstract

**Simple Summary:**

Lck is an important kinase that plays a key role in the physiological responses of T lymphocytes as well as in several other tissues such as the lung, intestine, brain, endometrium, and prostate. It can be found bound to the cell membrane, free in the cytosol, and even in the nucleus as it interacts with different proteins involved in various biological responses. Additionally, Lck is considered a proto-oncogene. Phosphatases and the Csk kinase control Lck activity: phosphatases can open and inactivate the structure, while Csk can only close it. The activation and modifications of Lck differ between normal and pathological conditions, such as cancer, allergy, autoimmunity, and graft vs. host disease. These differences may lead to new targets for pharmacological therapy. This overview briefly summarizes the characteristics and modulations of the enzyme.

**Abstract:**

Lck, a member of the Src kinase family, is a non-receptor tyrosine kinase involved in immune cell activation, antigen recognition, tumor growth, and cytotoxic response. The enzyme has usually been linked to T lymphocyte activation upon antigen recognition. Lck activation is central to CD4, CD8, and NK activation. However, recently, it has become clearer that activating the enzyme in CD8 cells can be independent of antigen presentation and enhance the cytotoxic response. The role of Lck in NK cytotoxic function has been controversial in a similar fashion as the role of the enzyme in CAR T cells. Inhibiting tyrosine kinases has been a highly successful approach to treating hematologic malignancies. The inhibitors may be useful in treating other tumor types, and they may be useful to prevent cell exhaustion. New, more selective inhibitors have been documented, and they have shown interesting activities not only in tumor growth but in the treatment of autoimmune diseases, asthma, and graft vs. host disease. Drug repurposing and bioinformatics can aid in solving several unsolved issues about the role of Lck in cancer. In summary, the role of Lck in immune response and tumor growth is not a simple event and requires more research.

## 1. Introduction

Lymphocyte cell-specific protein-tyrosine kinase (Lck) (EC 2.7.10.2), also referred to as Leukocyte C terminal Src kinase (LSK) or p56lck, is a transferase of the Src family kinases (SFKs) [1,2]. In vertebrates, this tyrosine-protein kinase family consists of eight additional members: subfamily SrcA Src, Yes, Fyn, and Fgr, and subfamily Src B Lck, Blk, Hck, and Lyn [2,3]. Their expression varies at the tissue and cellular level, and the variation also aligns with distinct hematopoietic lineages [4]. SFK’s role in initiating and regulating intracellular signaling pathways has long been recognized and attributed to tyrosine phosphorylation. This modification, by altering the protein conformation and yielding binding sites for Src homology 2 (SH2) domains and protein tyrosine-binding domains, allows for the regulation of protein activation and protein–protein interaction, respectively. Thus, this effectively enables the SFKs to function in positive and negative signaling cascade regulation [3,4,5].

Lck has been extensively researched in the context of T-cells, their thymic selection and maturation, signaling, and function, and their role in the B-cell lineage’s lymphocytic leukemia [3,4,5]. Nevertheless, Lck is also present in tissues [1]. The enzyme is highly expressed in the thymus and then in the appendix, which is higher than in the tonsils, the lymph node, and the spleen. Other tissues include the salivary gland, lung, small intestine, colon, gall bladder, esophagus, neurons, urinary bladder, prostate, and cervix [1].

Lck kinase is implicated in various oncogenic processes, particularly in colorectal cancer [6,7], chronic lymphocytic leukemia (CLL), and thymomas [8]. In colorectal cancer, Lck is expressed in some human colon carcinoma cell lines and is found at low levels in normal colonic epithelial cells, with its upregulation potentially leading to tumorigenesis. Lck inhibitors are therefore considered a promising strategy for treating colorectal cancer. In CLL, Lck promotes B-cell receptor (BCR) signaling, contributing to cell survival and glucocorticoid resistance and inhibiting Lck-inducing apoptosis in CLL cell lines. Targeting Lck in CLL is thus valuable for therapy. Additionally, in thymomas, abnormal expression of Lck can result in the proliferation of immature thymocytes, leading to tumorigenesis. Inhibitors designed to prevent Lck overexpression may be effective in treating thymomas. Beyond these cancers, Lck’s complex role in both immune response and cancer cell signaling, particularly in breast cancer [9,10], lung cancer [11], bile duct cancer [12], glioma [13], melanoma [14], and pancreatic endocrine tumors [15], presents a fascinating area for further research.

An analysis of Lck structure and function is essential to understanding the possibility of the enzyme and its modulation in cancer growth, therapy, and immune response. The manuscript provides a general view of the subject. A brief analysis of different inhibitors is also discussed.

## 2. Key Biochemical Elements of Enzyme Activity

Lck is primarily situated at the plasma membrane within the glycolipid-enriched membrane microdomains (GEMs), intimately connected to TCR-mediated signal transduction [16]. The GEMs are enriched in highly phosphorylated active forms of signaling components of TCR/CD3, T-cell receptor (TCR)–ζ chain-associated 70 kDa tyrosine phosphoprotein (ZAP-70), SH2-domain-containing leukocyte protein of 76 kDa (SLP-76), and phospholipase Cγ1 [17]. The activation of biochemical signaling pathways is triggered by TCR/coreceptor engagement, which governs the outcome of the T cell response in conjunction with signals from costimulatory molecules and cytokine receptors [17]. Lck is the first kinase involved in this process and a fundamental component of T-cell response. Although antigen presentation and TCR engagement are crucial for Lck activation, they are not essential, as was demonstrated in multiple studies where a small pool of Lck was found to be active without antigen presentation [5,6,7,8]; therefore, tight regulation of Lck activity is critical to maintaining physiological T-cell responses [8].

**Figure 1:**
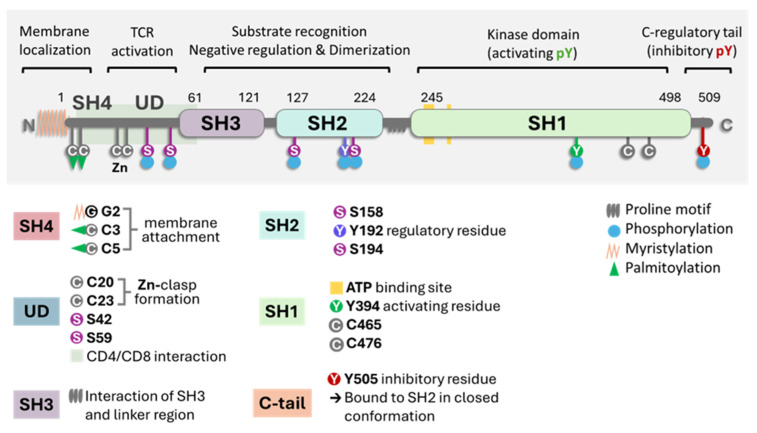
A general scheme of Lck with the molecule’s different domains and critical parts. The key points are the proline SH domains, the proline motif, the phosphorylation sites (S for serine and Y for tyrosine), and the link to the membrane (myristoylation or palmitoylation). In the closed state of the enzyme, it refers to phosphorylated at position Y505. Image generated using Bio Render software.

Figure 1 illustrates the critical parts of the enzyme structure [1]. The membrane-binding motif is essential for most antigen-dependent lymphocytes. The N-terminal Src homology 4 (SH4) domain is crucial for membrane anchoring. The domain SH4 is always myristoylated at glycine position 2 and can be reversibly palmitoylated [17,18,19,20]. Palmitoylation was found to be contingent on myristoylation. Lck is palmitoylated on Cys3 and Cys5 by zDHHC2 and zDHHC21 enzymes, which is critical for plasma membrane targeting and T-cell activation. The inhibition of the enzyme DHHC2 has been shown to inhibit the localization of Lck in lipid rafts [21]. PLC-γ1 and LAT also undergo S-palmitoylation, contributing to T-cell signaling and amino acids 7–35, which are the binding domains to CD4 and CD8α cytoplasmic tails [18,19]. Palmitoylation and myristoylation are increased in different oncogenic proteins since they enhance protein stability. Several pan inhibitors have been used in various tumors with partial success [22]. Specific enzyme inhibitors are being developed and analyzed in preclinical trials. The central question remains concerning these inhibitors’ effect on T lymphocytes’ normal cell function.

Since Lck is usually part of the lipid raft, a decrease in the cholesterol membrane prevents its incorporation, and consequently, T cell activation is reduced. Cholesterol contributes to T cell dysfunction by activating immune checkpoints and producing CD8+ T cell exhaustion [23,24,25]. Lowering cholesterol levels may disrupt immune checkpoint signaling and enhance immune checkpoint blockade immunotherapy. High cholesterol in the tumor microenvironment is linked to increased PD-1 expression in CD8+ T cells and CD8+ T cell exhaustion [24,25].

Protein lipidation is a fascinating field that has provided insights into different structures and metabolites involved in structural and biological functions [26]. The SH2 domain of Lck binds anionic lipids, which may modulate the enzyme’s activity [27,28]. However, the effect of omega-3 and omega-6 fatty acids and metabolites in this context remains unresolved. It has been suggested that membrane modifications with docosahexaenoic acid or eicosapentaenoic acid may affect the location of Lck in the lipid raft [29] and, consequently, enzyme activity.

Statins have been proposed as adjuvant therapy for different types of tumors [30,31]; however, a meta-analysis of clinical trials has not supported this hypothesis [31,32]. Benjamin DJ and coworkers [32] have suggested the effect of statins independently of cholesterol metabolism, although no precise mechanism has been discovered yet. Statins were shown to inhibit antigen presentation, modulate Th subpopulations, and decrease organelle traffic in vitro [30,31,32]. More studies are needed to understand the immunomodulatory role of statins and other lipid mediators.

In the critical membrane-related part of the enzyme, the SH4 domain, two cysteines are involved in Zn2+ binding to form a complex called the “zinc clasp.” This Zn domain seems critical for enzyme activity and T-cell responses [1,19,33]. The checkpoint inhibitor LAG-3 also competes with the Zn clasp of TCR and Lck [33]. As illustrated in Figure 1, the enzyme’s SH4 domain is followed by a unique domain (UD), domains SH3 and SH2, a short proline-rich linker region, and a catalytic (tyrosine kinase) domain SH1 preceding the short C-terminal tail. SH3 and SH2 domains regulate the catalytic domain’s conformational changes through proline-rich and phosphotyrosine-containing motifs. Thus, the phosphorylation of specific residues and the regulatory SH3 and SH2 domains control Lck activity [16,34,35,36]. Lck can be cis-phosphorylated by another molecule or trans-phosphorylated by another molecule of Lck [33,34]. There is the inhibitory C-terminal residue Y505, an activating residue in the kinase domain Y394; however, there are other phosphorylated residues, residue Y192, and 4 serine residues, S42, S59, S158, and S194 (Figure 1). Phosphorylation at Y192 maintains the structure open, while the serine S42 phosphorylation depends on PKA or PKC kinase activity and S59 on ERK activity [36,37]. The phosphorylated S59 residue seems involved in mitotic events leading to cell proliferation [37]. It is not clear what the roles of the other two serine residues are. The phosphorylated serines may be due to transphosphorylation [36]. In general, the activation mechanisms of Lck can vary depending on the specific signaling pathways and the cellular context.

Wu et al. [38] observed that asparagine directly binds to Lck and functions as a signaling molecule in CD8+ T cells. They reported that the Asn binding site in the Lck structure lies within the catalytic domain (residues 240–320). Asn favors Y394 over Y505 phosphorylation, promoting T-cell activation and memory response [38]. A similar effect on regulatory residues was reported in disulfiram (DSF), which was repurposed and enhanced the anti-tumor immunity of CD8+ T cells [39]. DSF, as a Zinc finger active compound, interacts with Lck through a direct covalent bond via Cysteine 20/23 residue and promotes Y394 phosphorylation, elevating Lck activation and immobilizing active Lck [39].

**Figure 2:**
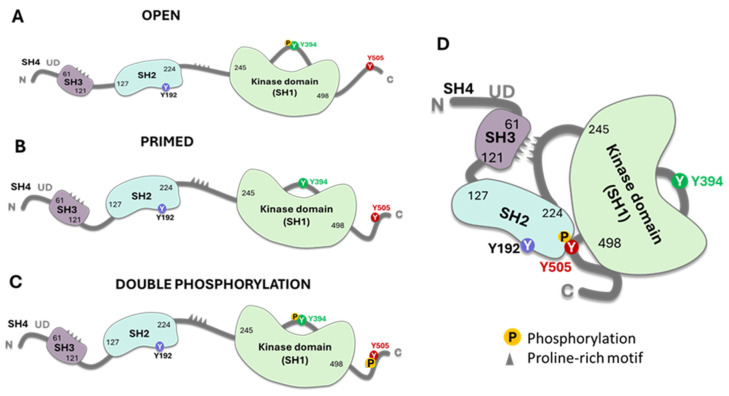
The model represents the different conformational and functional models of Lck. The open structure (**A**) shows the kinase active domain phosphorylated at Y394. This structure differs from the primed structure (**B**) in which the enzyme has an active conformation, but the Y394 residue is not phosphorylated. However, this structure can be cis or trans-phosphorylated. The open structure may have the residue Y192 phosphorylated (see Figure 3 for the physiological relevance). (**C**) Double phosphorylation of Y394 and Y505 is possible, rendering an active enzyme that differs from the closed state on the right (**D**). It is unclear if the enzyme can be phosphorylated in the three tyrosine residues or if serine phosphorylation affects the kinetics of the active conformation. This figure was created using Bio Render software (https://www.biorender.com/, accessed on 21 July 2024). For 3D structure conformation, consult Prakash D et al. [47].

The enzyme’s activity in physiologic conditions depends on CD45 and Csk. The role of the phosphatase CD45 and the Csk kinase on Lck activity has been studied in detail [40,41,42]. CD45 causes Lck to unfold via dephosphorylation of Y505, allowing for the trans-autophosphorylation of Y394, leading to the full activation of Lck [35,41]. Its activity is not regulated through the phosphorylation of its kinase domain [35,41]. Instead, it is allosterically activated when its SH2 domain interacts with SFK-phosphorylated motifs [35,41]. CD45 can dephosphorylate tyrosine residues in C-terminal Y505 and, in the kinase domain, Y394, and its activity is > 20-fold faster than Lck in an in vitro reconstitution study documented by Hui and Vale [43]. However, Lck phosphorylation on the SH2 domain at Y192 decreases the regulatory effect of CD45 on Lck [44,45,46], implying that Y192 phosphorylation regulates Lck activity independently of CD45. Courtney and co-workers [45] have shown negative feedback of phosphorylated ZAP70 on Lck due to the phosphorylation of Y192. The phosphorylation renders Lck resistant to CD45 dephosphorylation but unable to be properly activated. The critical issue is that the Lck structure could be open independently of CD45, and the CD45-resistant phosphorylation impairs T-cell response (Figure 2).

The Csk kinase catalyzes the transfer of the phosphate group to the Y505 residue that interacts with the SH2 domain and enables the SH3 domain to engage with the proline-rich linker region; the enzyme goes to a “closed” inactive state [42]. The Csk kinase can bind to phosphatases PTPN12 (PTP-PEST) and immune-cell PTPN22 (LYP/Pep) with the SH3 domain, which promotes autoinhibition by dephosphorylating the activation loop [48]. As a cytosolic protein, Csk lacks membrane-anchoring lipid-binding sites. This raises the need for a Csk-binding protein recruiter to ensure its localization at the plasma membrane for effective negative regulation of Lck activity [42,48]. These proteins include Csk-binding protein/phosphoprotein associated with glycosphingolipid-enriched microdomains (CBP/PAG1), Fak, Paxillin, and the Dok family [42,48,49,50]. Once it is open, the likelihood of Y505 trans-autophosphorylation/phosphorylation is low, suggesting that phosphatases are crucial in closing the structure [42,48,49].

Two adaptor proteins, LIME (Lck interacting molecule) and PAG (glycosphingolipid-enriched microdomains), have been involved in Csk inhibition of Lck activity [50,51,52]. LIME is involved in the inflammatory response [50]. The putative mechanism of Lck negative regulation/inhibition stems from PAG1 phosphorylation loss upon TCR stimulus while being abundantly phosphorylated in unstimulated T cells, which allows Csk to dissociate from PAG1/engage with Lck at the membrane, respectively [52]. Both adaptor proteins serve as membrane anchors for the Csk enzyme, facilitating the effect of Csk on the C terminal of Lck and the closure of the enzyme. As depicted in Figure 4, the adaptor proteins recruit Csk in both cases, which is responsible for phosphorylating Lck. The critical issue is the binding of Csk to the phosphorylated residues of both adaptor proteins. Csk interaction with Lck may be modified depending on the interaction with other kinds of kinases depending on the SH2 and SH3 domains.

**Figure 3:**
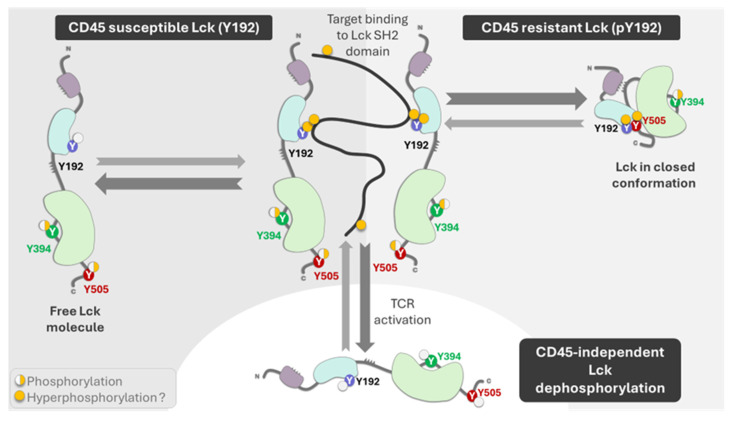
The figure depicts the role of CD45 and other phosphatases on Lck phosphorylation at residue Y192. This figure was modified from the proposed model described by Borowicz et al. [53]. The phosphorylation can be dependent (susceptible) on CD45 dephosphorylation, or it can be resistant to phosphorylation and maintain the enzyme in close conformation. The thick lines correspond to the preferential function of the enzyme, the physiological response. Other phosphatases can dephosphorylate residues Y192, Y394, and Y505 to generate a nonfunctional enzyme. The previous model differed in its assessment of the importance of Tyr192 for TCR signaling, the hyperphosphorylation of the Tyr192 residue, the role of free Lck, and the role of other phosphatases. This figure was made using Bio Render software.

**Figure 4:**
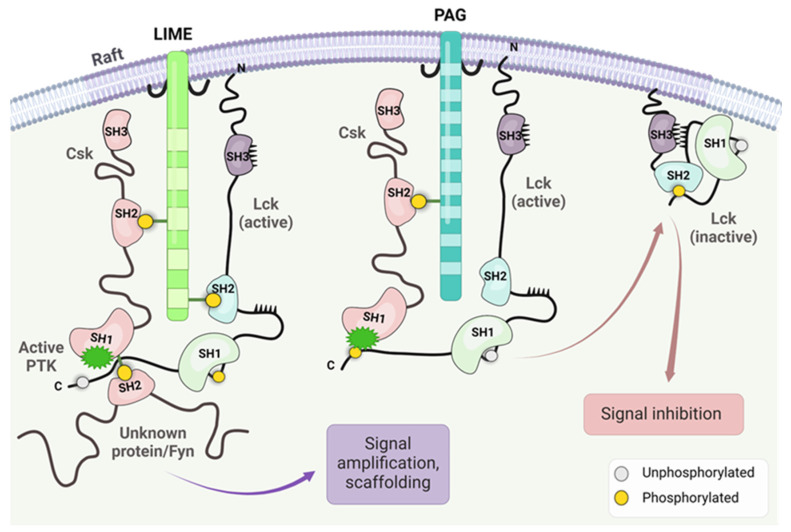
The figure illustrates the importance of the adaptor proteins on Csk anchoring and Lck interaction with Fyn and other kinase and Csk-induced closure of the Lck open structure. The effect of the interaction of Csk linked by phosphorylated residue on the SH2 domain of PAG or LIME generated two different physiological responses. The figure was made using Bio Render software.

In severe combined immunodeficiency, ZAP70 cysteine 564 seems to have a regulatory function and is involved in TCR and Lck activation [54]. Cysteine residues outside the catalytic domains of protein tyrosine kinases are critical for enzyme activity. Reversible modifications such as nitrosylation, sulfonation, and glutathionylation have modulated kinase activity [55,56,57,58]. These modifications can be crucial in enzyme catalytic activity and in enhancing alterations in cell metabolism, leading to malignancy [59].

**Figure 5:**
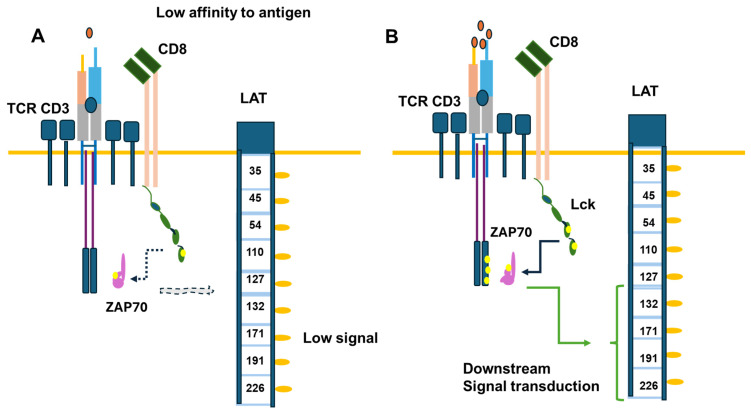
Signal transduction induced in CD8 upon antigen binding. Part (**A**) of the figure illustrates the response induced by a low antigen level compared to a normal level in part (**B**) of the Figure. The critical element is Lck involvement in TCR γ chains, the phosphorylation of ZAP70, and the phosphorylation of the adaptor protein LAT, activating signaling pathways downstream. The yellow circles refer to phosphorylated tyrosine.

Signal transduction is induced upon antigen binding to the CD3/TCR in CD4 or CD8 cells in normal physiological conditions [58]. The generation of signal transduction upon Lck activation and the involvement of TCR phosphorylation, ZAP70, and the adaptor protein LAT have been extensively reviewed [58]. New elements indicate that the enzyme may be more active when not attached to the membrane [60,61]. The enzyme’s activity differs in different T-cell subpopulations [61]. The regulatory T cells were shown to have less active Lck than committed T helper or cytotoxic cells [62]. Moreover, with age, Lck activation through co-receptors may decrease. Co-activating molecules like CD28 may play an important role in this effect [63,64]. Also, cytomegalovirus infection has decreased CD8 activity by affecting CD28 signaling [65], and mice lacking CD28 have higher cytotoxic responses [66]. In the T cell receptor activation dynamic, Lck is more active in CD8 mature cells than CD4 mature cells, suggesting that Lck activation is critical in the CD8 cytotoxic response [61,67]. Figure 5 illustrates the effects of antigen recognition, Lck activation, and the dynamics of signal transduction. Figure 5 only represents a partial process in which Lck is involved. The coreceptors can also recruit Lck and other kinases to amplify the antigen-induced signal. On the other hand, CTLA-4 recruits the phosphatase SHP-2, which similarly dephosphorylates Lck as PD1 [59,60] (Figure 5).

Lck activates Erk through phosphorylation in two distinct ways. Firstly, the activation of Erk is increased and maintained when TCR is induced in the presence of certain antioxidants like NAC, MnTBAP, and Ebselen, which inhibit peroxide production. This increase in Erk phosphorylation is linked to higher serine phosphorylation of Lck, showing that ROS acts before MEK-ERK activation [68]. Also, targeting peroxide by overexpressing peroxiredoxin II further supports this mechanism [68]. Secondly, when T lymphocytes are stimulated with calcium ionophores, Erk1 and Erk2 are phosphorylated and activated through a calmodulin and CaM-kinase pathway. This calcium-induced Erk activation depends on the presence and phosphorylation of Lck, as demonstrated by experiments with Lck-negative and reconstituted cell lines. Inhibiting Lck with PP2 blocks calcium-induced, but not PMA-induced, Erk activation, confirming the role of Lck in this signaling cascade [68,69]. Therefore, Lck activates Erk through ROS-dependent modulation and calcium/calmodulin-mediated phosphorylation pathways.

Proteins involved in protein and cell traffic may also be related to the aggrupation of Lck in the initial part of cell activation. Nexin 27 has recently been shown to induce the highly efficient CD4 TCR signal complex associated with Lck [70]. Even though nexin 9 has been associated with CD8 cell exhaustion [71], the role of other nexin proteins in T cell physiology is still unresolved. Nonetheless, nexin proteins have been associated with cholesterol transport and may be crucial in redirecting Lck in the membrane function of T cell lymphocytes [72].

In activated T cells, c-Cbl, a ubiquitin ligase, explicitly targets and degrades the proximal activating cytoplasmic tyrosine kinase Lck and the adaptor protein LAT, which is essential for TCR signal transduction [73,74]. Depletion of c-Cbl results in a defect in TCR-mediated LAT internalization and elevated levels of LAT within T cells [74]. Additionally, c-Cbl is implicated in T cell anergy by interacting with the CrkL adaptor protein and C3G, the guanine nucleotide exchange factor for Rap1 [75,76]. However, c-Cbl was found to induce PD1 degradation, suggesting a dual role [77]. Cbl-b is also an essential target for tumor therapy since it blocks T cell activation by TCR and CD28 involving Lck function [78,79].

Other ubiquitin 3 ligase proteins involved in Lck degradation are SOCS-6 and SOCS-1 [80]. Moreover, the E3 ubiquitin ligase UBR2 directly activates Lck by phosphorylating Y394 and ubiquitinating the protein at Lys99 and Lys276. However, when the phosphatase DUSP22 dephosphorylates UBR2 in specific serine residues, it induces the degradation of the ligase, leaving Lck ubiquitinated [81]. This proposed mechanism explains the low ubiquitinated Lck in T cells in Lupus erythematosus patients [82]. The control of Lck’s ubiquitination may facilitate the decrease in active proto-oncogenic enzymes.

**Figure 6:**
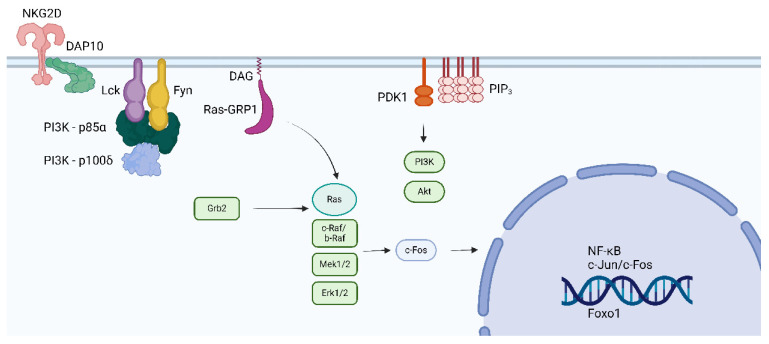
The figure depicts the interaction between the killing receptor NKG2D (CD314) and Lck and Fyn. The interaction is dependent on DAP10 and DOC8. CD314 is expressed in NK and CD8 cells. Phosphorylation of Lck by the WASP prevents the cytotoxic response of NK and CD8 cells [45]. The figure was made using Bio Render software.

The complex DAP10/Syk plays a major role in NK-induced cytotoxic activity. However, the Wiskott–Aldrich Syndrome protein (WASP) phosphorylates Lck at position Y141 and inhibits enzyme activity and NK cell cytotoxicity, suggesting that Lck, besides Syk, is involved in cytotoxic responses independent of TCR and CD/CD8 antigens [83]. Figure 6 illustrates the possible link between Lck and NKG2D receptors for NK cell signal transduction involved with cytotoxicity and the role of Lck in the induction of CD314.

SHP-1 and SHP-2, recruited by inhibitory receptors, are key negative regulators of proximal TCR signaling. They can dephosphorylate multiple crucial cascade elements, including Lck (on the Y394 residue), TCR ITAMs, and ZAP-70 [84,85,86]. The PD-1 (CD279) receptor ligation leads to PAG1 tyrosine-phosphorylation and recruitment of Csk or the phosphorylation of SHP-2, which dephosphorylates Lck and inhibits it [84,85,86]. The interactions are critical for cancer chemotherapy [87,88]. Posttranslational modifications may be important in finding new therapeutic targets [89]. In activated T cells, Lck was co-immunoprecipitated with the TRAIL-R/SHP-1 complex [89]. The involvement of TRAIL led to the interruption of proximal TCR signaling, as it hindered the Y394 Lck phosphorylation and dampened the Lck recruitment to the lipid rafts [90]. Thus, TRAIL-R could be a new type of immune checkpoint receptor that can limit T cell activation. The TRAIL-R/SHP-1 axis could be a potential target for treating immune-mediated diseases.

TAOK3 is a kinase involved in anti-microtubule drug resistance in mammary tumor cells via NFkB [91]. Recently, the role of the kinase TAOK3, which belongs to the STE-20 family, has been revealed in lymphocytes [92]. Its absence reduces T-cell receptor signaling and increases the interaction of the tyrosine phosphatase SHP-1 with the kinase LCK [92]. Thus, complex protein interactions differ between Lck membrane-bound and cytosol, and different phosphatases can modulate the control of enzyme activity or the signal transduction induced by Lck.

**Figure 7:**
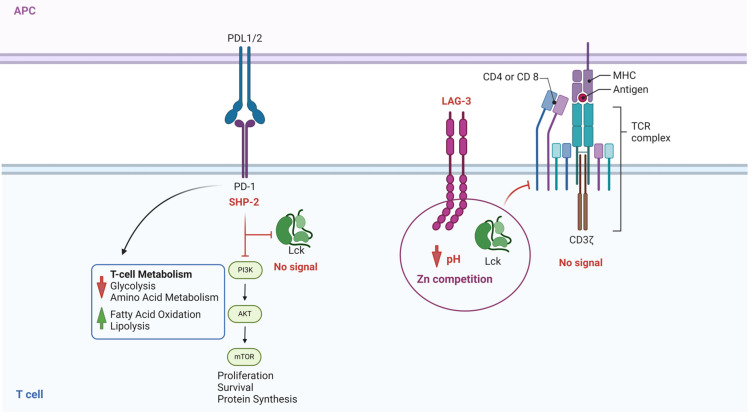
Role of the Inhibitor Receptors on Lck activation. This figure illustrates two different processes. On the left, SHP-2 activation induced by PD-1/PDL-1/2 ligation prevents the phosphorylation of Lck and, consequently, the signal generated by TCR/ZAP70/LAT phosphorylation. On the right side, the figure illustrates the effect of LAG-3, the intracellular part of the protein that competes for Zn and blocks the interaction of the Zn domain on Lck and CD4/CD8 receptors, not allowing the enzyme to be activated. Inhibition of PD1/PDL1/PDL2 and LAG-3 (checkpoint inhibitors) impedes the inhibition of Lck and restores the signal transduction required for a proper T-cell response. The figure was made using Bio Render software.

Figure 7 illustrates checkpoint inhibitors’ effects on two different inhibition types: the interaction of PD1/PDL-1 or PDL-2 and the inhibitory receptor LAG-3 [93]. LAG-3 is expressed in exhausted cells and binds to the TCR CD4/CD8 cluster, decreasing the pH and binding to the zinc pocket [94]. LAG-3 can bind to different glycan structures, including galectin and α synuclein [93], suggesting it could be an essential modulator of tumor-infiltrating lymphocytes [94]. The inhibition of the LAG-3 signal through antibodies has been suggested for therapy, and bi-specific antibodies against PD1 and LAG3 were postulated to have a broader effect in hematological and solid tumors [95].

Lck has been shown to bind to CD2, CD5, CD44, CD48, CD55, CD146, and CD160 [1]. These receptors are involved in several biological responses. CD2/LFA-3 is an interesting complex critical to immunological synapses, and CD2 is preferentially expressed in memory cells [96]. CD2 interacts with Lck and Fyn, forming the lipid raft crucial for T-cell response [97]. CD5 can also be phosphorylated by Lck and is involved in lymphocyte signal transduction, particularly in B lymphocytes [98]. The role of Lck in B cell malignancies is noted but difficult to analyze due to the low proportion of the kinase compared to Btk. It would be interesting to analyze possible scenarios for the role of Lck in B cell physiological responses.

CD44 is a cell surface adhesion receptor upregulated in primed naïve T lymphocytes and highly expressed in T memory cells [99]. Lck is bound to the intracellular domain side of the receptors, which can be triggered by hyaluronic acid and has less affinity with other extracellular matrix glycosaminoglycans and proteoglycans [99]. It is also highly expressed in tumors, cancer stem cells, and metastatic sites [100], and different isoforms may have other functions in cell physiology and pathology [101]. CD44 expression in tumor cells could be related to poor survival; however, there are still controversies about the receptor and the possible role of Lck in cell migration and physiological responses.

CD146 (MCAM-melanoma cell adhesion molecule), a cell surface adhesion molecule for Laminin 411, has also been related to Lck activity. In mouse T cells, CD146 deficiency impairs thymocyte development and peripheral activation. CD146 interacts with Lck and can be found in monomeric and dimeric forms in T cells, with the dimerized form increasing after TCR ligation. Dimerized CD146 recruits Lck and promotes Lck autophosphorylation. In tumor models, CD146 deficiency dramatically impairs the antitumor response of T cells [102]. T cells expressing CD146 in humans are mainly responsible for IL-17 production [103]. Inhibition of Lck in the psoriatic mice model has shown improvement [104]. These results generate new elements in which free Lck can bind to different receptors and activate signal transduction signals dependent on the vicinity of other kinases and proteins, mainly through the SH2 and SH3 domains, but also with the zinc pocket in the SH4 domain, which may allow the protein to interact independently of the membrane anchors and facilitate lymphocyte activation, T and NK cell.

CD48, a member of the SLAM family of proteins, is a known B and T cell marker involved in cell activation and interaction [66]. The molecule is also expressed in macrophages at inflammatory sites. CD48 can interact with CD2 or CD244 [105]. CD48 cooperates with Lck in the lipid raft, but CD2 may also facilitate the cis interaction of the kinase in the CD2:CD48 binding [106], but also in CD2:CD244 stimulation, leading to a reasonable supposition that Lck can be involved in physiological and pathological responses based on receptor expression. Also, in the immune synapse, it is difficult to conceive which interaction would be prevalent in priming, activating, or maintaining T cytotoxic responses against tumor cells.

The decay accelerating factor (CD55) is involved in the complement pathway, but it is also a potent activator of naïve CD4 T lymphocytes and is involved in tumor cell growth [107]. The interaction between CD55 and Lck seems to depend upon the lipid rafts formed upon cell binding, which may predispose the enzyme to the interaction with the adaptor protein LIME or the counteraction with the orphan receptor ROR2. Inhibition of Lck sensitizes endometrioid tumors to cisplatin [108]. A similar mechanism can probably be proposed for other epithelial tumors where cell interaction is vital for chemoresistance.

A vital cell marker for CD8 and NK cells related to cytotoxic efficiency is CD160 [109]. There are two isoforms, one transmembrane and another linked to the membrane by GIP. Lck has been linked to the transmembrane form of CD160 [109]. The antigen is also expressed in B-cell hematologic and mammary tumors [110]. It has been indirectly proposed that this interaction in tumors can lead to new therapeutic strategies [111,112]. However, it is important to note that Lck inhibition has decreased metastatic potential and invasion in oral cancer.

**Figure 8:**
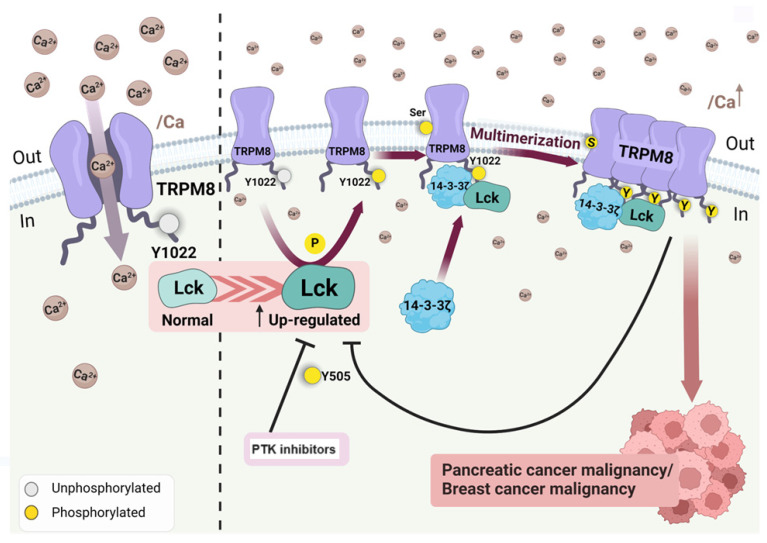
The figure illustrates the interaction between TRPM8, a calcium channel, and the activity of Lck. Lck phosphorylates the Y1022 residue, which, in the presence of 14-3-3ζ, forms a multimeric complex in pancreatic and mammary tumor cells. Protein tyrosine kinase inhibitors, saracatinib, and dasatinib can block this event. Pre-treatment with PTK inhibitors generates susceptibility of the tumor to cisplatin. Tumors that express Lck can be targeted by the PTK inhibitors, enhancing tumor susceptibility to standard treatment. The figure was modified from Huang Y et al. [119]. The Bio Render software was used to make the figure.

In addition to the above receptors and proteins, Lck also binds to other effector molecules such as VAV1, RASA1, and FYB1 and protein kinases, including AXL, RAF1, Fyn, Syk, and PI3K, via its SH3 domain and to the tyrosine-phosphorylated form of KHDRBS1/p70 via its SH2 domain. These bindings highlight the non-unique role of Lck in cell physiological responses. Figure 8 highlights the effect of Lck activity on multimerization of the Calcium channel transporter TRPM8 in pancreatic and mammary cancer [113]. It is unknown whether the up-regulated Lck escapes from normal physiological control; the specific Lck inhibitors may facilitate, as in other tumors, the effect of other drugs to enhance tumor clearance. Modulation of Lck activity entails an intricate interplay of membrane localization, phosphorylation, protein–protein interactions, and the influence of supporting molecules.

Lck is associated with the migration to the nuclei of the Ras protein RASSF5, which is involved in cell growth and tumor suppression [114,115,116]. Lck’s domain SH2 is engaged in the process. On the other hand, Vahedi and coworkers were the first to show that Lck is present in the nuclei of leukemic cells [114]. Lck is activated in nuclei and is involved in DNA damage repair by stabilizing RAD51 and BRCA1 in chemo-resistant ovarian cancer [115,116]. Lck’s presence and function may be similar in other tumors and can be an important therapeutic target [117]. New insights in NfkB signaling and Lck phosphorylation have been reviewed and, interestingly, give a perspective on cell activation and enzyme localization in tumor biology [118]. The role of NFkB inhibitors on Lck is still under research.

## 3. CAR T Cells and Lck Signaling

In CAR T signaling, as in normal CD8 function, TCRs and the recruitment of Lck play a major role in the response of activated CAR-T [120]. Constructing suitable receptors and coreceptors involves Lck, Fyn, and Syk enzymes to potentiate cell activation [120]. In addition, CAR T cells expressing NKG2D antigens are successful in pancreatic cancer. The CAR-T-modified cells decreased the stroma cells surrounding the pancreatic tumor, exposing the neoplastic cells to immune cells and rendering them more susceptible to cytotoxic killing in preclinical models [121,122,123]. Interestingly, CAR T NKG2D cells have been used in preclinical models to eliminate cell senescence, decreasing the response pack. It is unclear whether the induction of exhaustion markers on CAR-T cells is due to continuous cell activation or the absence of stimulating factors.

The partial success of the other CAR T clinical trials depends on tumor heterogeneity. Acarya S and coworkers [124] recently published the importance of CD28 increasing NK cytotoxicity through Lck/CD3Z/ZAP70, contrasting with previous reports that Fyn kinase signaling in CAR T cells was more efficient than Lck signaling [125]. Probably, the difference in the construct in which THEMIS, an activator of SHP phosphatase, is recruited is responsible for the effect [126]. More research may involve properly generating constructs to enhance the effectiveness of the treatment.

It is essential to analyze the role of previous protein interaction; ubiquitination, phosphorylation, and dephosphorylation may be modified therapeutically to enhance CAR-T and probably CAR-NK efficiency against hematologic tumors and solid tumors and decrease cell exhaustion.

## 4. Lck Genetic Analysis

The first evidence of the Lck mutation and lack of protein function came from Goldsmith and Weiss [127], who described the cell Jurkat variant Jcam, which lacks functional Lck due to a deletion in exon 7. However, Oh-Hori and colleagues [128] observed a lack of Lck activity in the T cell line infected with HTLV [128]. Data generated using those cell lines were influential in the T-cell signaling analysis and how the cell activation process was impaired with mutations.

Hauck et al. [129] reported a case of a patient with a homozygous missense mutation in the *Lck* gene, resulting in undetectable kinase activity and impaired T-cell receptor (TCR) signaling, leading to severe combined immunodeficiency (SCID) characterized by recurrent infections and poor T-cell function [129]. Similarly, Lanz et al. [130] described a biallelic *Lck* mutation in a patient with profound T-cell immune deficiency. This mutation affected the protein’s stability and function, leading to defective TCR signaling and reduced expression of co-receptors CD4 and CD8, resulting in severe infections and immunodeficiency, highlighting the critical role of Lck in the immune response [130]. Nevertheless, Lui and coworkers [131] analyzed a novel *Lck* variant (c.1318C>T; P440S) characterized by T cell lymphopenia with a skewed memory phenotype and infant-onset recurrent infections. The partial defect in *Lck* caused T-cell immunodeficiency and intestinal inflammation [131]. The quantity of transcribed enzyme was enough to allow the maturation of some conventional T cells but not regulatory T cells, which led to intestinal inflammation. Thus, there are probably more *Lck* mutations involved in tissue dysfunction and impaired immune responses.

After analyzing the documented structure of the gene, more than 20 SNPs were reported [1]. However, two main rs2124355238, which includes a frameshift AC/A variant deletion Ala160fs, and the rs587777335 variant that has eight missense sub-variants T/C Leu/Pro 2 at position 290. These variants have been analyzed in type 1 diabetes [1]. However, there are other polymorphisms: two at position 341, two nonpathogenic, one at position 348, one at position 385, and two at position 399. Also, three in the promoter region promoters Lck1-3 and four in the region bind to LIME 1-4 [1].

The functional consequences of *Lck* variants can be significant, impacting the protein’s stability and activity. For example, the C465R mutation disrupts hydrogen bonding within the kinase domain, leading to reduced protein expression and stability. Experiments have confirmed that this mutation affects TCR signaling pathways by causing deficient protein phosphorylation in T-cells expressing the mutant *Lck* [132]. *Lck* gene polymorphisms were studied concerning type 1 diabetes (T1D). Hulme et al. [133] identified seven significant single nucleotide polymorphisms (SNPs) but concluded that common *Lck* polymorphisms are unlikely to play a substantial role in T1D susceptibility. In contrast, Zhu et al. [133] found a significant association between the SNP rs10914542 in the *Lck* gene and T1D. The G allele of rs10914542 was linked to reduced T-cell activation and increased T1D susceptibility [134].

Since the expression of *Lck* differs in several tumors, it is unclear what mechanism controls the enzyme’s transcription, translation, post-translational, and post-transcription changes. A comprehensive analysis has been published [135]; however, bioinformatics and artificial intelligence analysis of the data suggest that an *Lck* metagene analysis (a group of genes associated with *Lck* expression and function) can be beneficial in cancer prognostics based on the published favorable prognosis of the cluster in breast cancer patients [136]. There is a need for standardized clinical trials in which proper data analysis with relevant patient information may provide new avenues of research.

## 5. Pharmacological Modulation of Lck Activity

Protein kinase inhibitors (PKIs) are crucial in treating various cancers and inflammatory conditions. The list of inhibitors that can affect Lck catalytic activity among other kinases includes dasatinib, imatinib, bosutinib, ponatinib, masitinib, ceritinib, crizotinib, fostamatinib (used on thrombocytopenia), tribanibulin (inhibit tubulin), and sacaritinib (now proposed for non-cancer therapy), as well as the experimental drugs A-770041 and WH-4-23. Even though most inhibitors are not specific, pan tyrosine kinase inhibitors have changed the landscape of targeted cancer therapy [137,138,139,140,141,142], and the structures and main biological effects have been documented [137,138,139,140,141,142]. As described earlier, several solid tumors have high expression of Lck, and inhibiting the kinase activity can make the tumor more susceptible to other anti-cancer drugs.

A prediction model for Lck inhibition has been developed, and it is reasonable for assessing enzyme activity in different models based on the structures [142]. The development of new compounds should probably focus on the enzyme’s kinase activity, membrane anchor, and zinc pocket elements in the SH4 domain.

Imatinib is used in malignant gastrointestinal stromal tumors (GIST) [141,142,143,144,145]. Imatinib plus binimetinib (a MEK inhibitor) were used in the first-line treatment of advanced GIST [146]. Nilotinib is active in KIT-mutant metastatic acral and mucosal melanoma [146]. Bosutinib and pemetrexed (antifolate drug) were administered in patients with advanced metastatic solid tumors (adenocarcinoma of the lung, adenocarcinoma of the appendix, and urothelial carcinoma) that had progressed on “standard of care” chemotherapy in a phase I study with some success [146]. In patients with advanced pancreatic ductal adenocarcinoma and overexpression of acyl-CoA oxidase-1 (ACOX1), gemcitabine plus masitinib showed a better OS in comparison with gemcitabine plus placebo [147]. Median survival was significantly longer for patients with advanced imatinib-resistant GIST receiving masitinib, followed by post-progression addition of sunitinib when compared against patients treated directly with sunitinib in second-line [147,148]. Also, masitinib is effective as a first-line treatment of advanced GIST with comparable results to imatinib regarding safety and response [149]. Promising results were observed using nilotinib in KIT-driven advanced melanoma, a condition with very few therapeutic options [113,150]. Moreover, Lck was shown to be involved in invasion and metastasis in oral cancer, and the specific inhibitors can be a useful therapy in these patients [151]. In summary, several studies emphasize the effect of tyrosine kinase inhibitors in different solid tumors, in which Lck may play an important role. The question also arises of whether the structures have other biological functions that could be used for treatment. As an example of drug repurposing, Barwal A and coauthors showed that ponatinib could bind to PD-1 and inhibit the interaction with PDL-1/2 [152]. There is still room for improvement in cancer therapy using these inhibitors.

Concerning the new structures, A-770041 has been found to inhibit CTV-1 cells (an acute myeloid leukemia cell line) [153], and treatment of human glioma stem cells with A-770041 has crizotinib, resulted in significant inhibition of self-renewal and tumor-sphere formation [154]. WH-4-23, a potent inhibitor for Lck, also inhibits CTV-1 cells [153]. Crizotinib is a TKI that potently inhibits Lck, anaplastic lymphoma kinase (ALK), mesenchymal–epithelial transition (MET), and ROS proto-oncogene 1, receptor tyrosine kinase (ROS1) [153,154,155]. Crizotinib is well tolerated with rapid and durable responses in patients with ALK-positive non-small-cell lung cancer [156] and is superior to chemotherapy [114]. Tanaka A and coworkers [157] showed that imatinib selectively depleted effector T reg (eT reg) cells and significantly increased effector/memory CD8+ T cells, enhancing the antitumor response. New structures or drug repurposing can enhance therapeutic efficiency in solid tumors.

It is also essential to address the issue that Lck inhibitors may be beneficial for treating immune-related disorders and not only as an anti-tumor treatment. Several T-cell-dependent responses can be inhibited, such as autoimmunity, inflammatory diseases, and organ transplant rejection [158,159,160,161,162,163,164,165]. This effect could be due to FoxP3-dependent much lower expression of Lck and ZAP-70 in T reg cells compared with other T cells. Imatinib inhibition of Lck further reduced their TCR signal intensity, rendering them selectively susceptible to signal-deprived apoptosis [158]. Other inflammatory diseases, such as lung fibrosis, asthma, rheumatoid arthritis, and diabetes, can be treated with Lck inhibitors [160,161,162]. On the other hand, calcineurin inhibitors target Lck activation in graft vs. host disease [163], generating questions concerning treatment effectiveness and selectivity.

Nilotinib in patients with cGVHD steroid-dependent/refractory cases was associated with a substantial decrease in proinflammatory cytokines, which improved the outcome of bone marrow transplant [163,164]. Moreover, Nilotinib has been used to prevent cytomegalovirus (CMV) infection in patients with allogeneic hematopoietic stem cell transplantation by blocking platelet-derived growth factor receptor-alpha, a critical receptor for this virus [165,166,167].

Table 1 illustrates the different inhibitors that have been developed. The structures were designed for specific targets; however, the compounds can inhibit more than one target with different IC50s. Most structures are from compounds already used in therapeutics or advanced clinical trials. However, several structures under development are currently being tested in preclinical studies.

## 6. Conclusions

**Figure 9:**
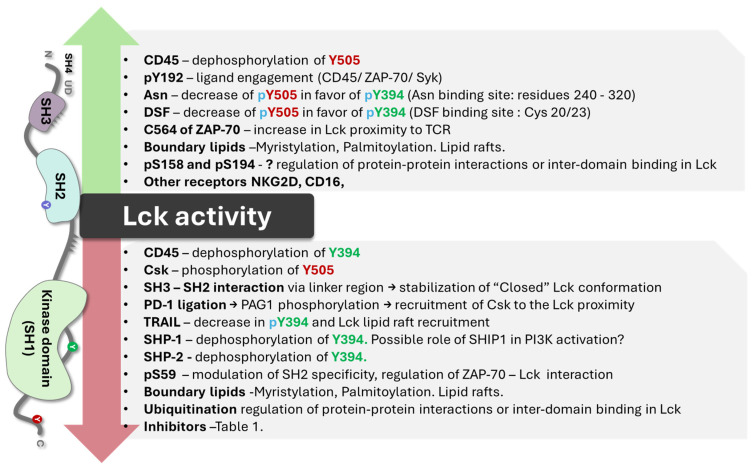
Represents an overview of compounds and events that affect Lck activity.

The role of Lck in the adaptative immune response has been studied; however, there are still questions concerning the importance of enzyme activity in specific subpopulations and the importance of the sequence of events involving immune synapsis, the recruitment of membrane-free or bound enzymes, and the role of Lck activation and inhibition by protein–protein interaction aside from CD45 and Csk. This information is crucial in understanding the immune response against tumors and how its Lck modulation can lead to a practical cytotoxic function, T cell exhaustion, or the generation of T regulatory cells. Interesting but unsolved questions have been observed in CAR-T and CAR-NK cells, as well as cell cytotoxic function and exhaustion, which need to be resolved. Figure 9 summarizes the regulation of Lck activity in different conditions. Since lipid rafts and modifications modify the enzyme’s location and function, only partial information has been found in some items, like boundary lipids.

On the other hand, the role of Lck in cancer is complex. In hematological tumors, the role of Lck and other kinases is directly linked to tumor growth. In solid tumors, progression and metastasis have been related to Lck activity. However, Lck overexpression in solid tumors is related to increased survival, and treatment with pan TK inhibitors or Lck inhibitors is crucial for the increase in susceptibility to standard treatments, showing that the enzyme activity and not only its presence can be involved in tumor growth.

New interesting tyrosine kinase inhibitor molecules have been developed that show promise for cancer treatment. Lck inhibitors may also modulate T-cell responses in acute and chronic inflammatory responses. Drug repurposing and chemical modifications of known structures can open new avenues of research for understanding disease mechanisms and new therapeutic targets for more efficient and specific effects.

## Figures and Tables

**Table 1:** Inhibitors of the Lck.

Inhibitor	Structure	Site of Binding	Initial Proposed Target	References
Saracatinib	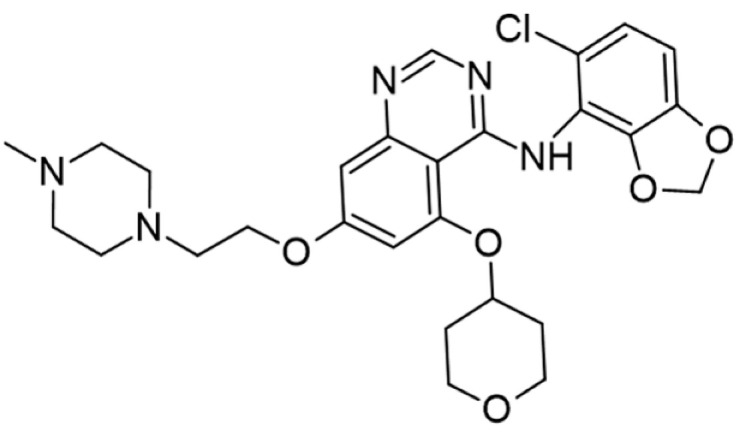	ATP-binding site	Selective Scr inhibitor	[137,138,139]
Dasatinib	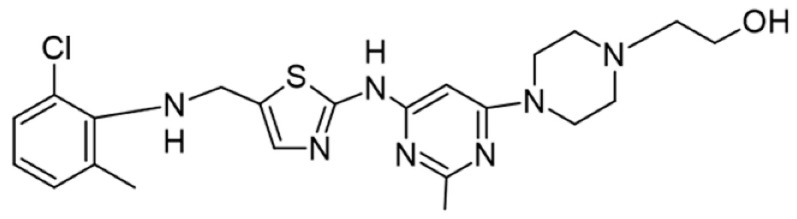	Tyr-394	ABL kinase domain	[137,138,139,141]
Imatinib	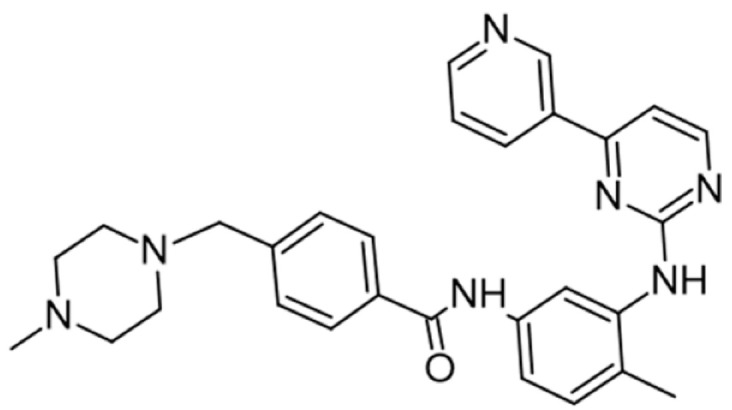	ATP-binding site	Pan tyrosine kinase inhibitor	[137,138,141]
Bosutinib	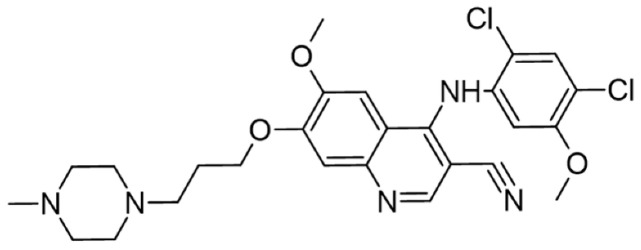	ATP-binding site	BCR-ABL inhibitor	[13,14,15,16,17,18,19,20,21,22,23,24,25,26,27,28,29,30,31,32,33,34,35,36,37,38,39,40,41,42,43,44,45,46,47,48,49,50,51,52,54,55,56,57,58,59,60,61,62,63,64,65,66,67,68,69,70,71,72,73,74,75,76,77,78,79,80,81,82,83,84,85,86,87,88,89,90,91,92,93,94,95,96,97,98,99,100,101,102,103,104,105,106,107,108,109,110,111,112,113,114,115,116,117,118,119,120,121,122,123,124,125,126,127,128,129,130,131,132,133,134,135,136,137,138,147]
Ponatinib	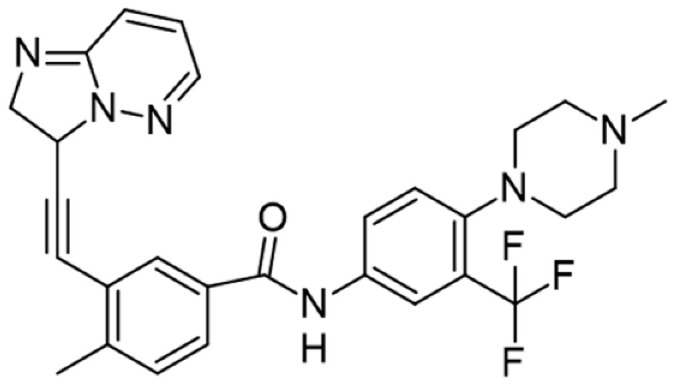	ATP-binding site	ABL kinase inactive site	[137,138]
Masitinib	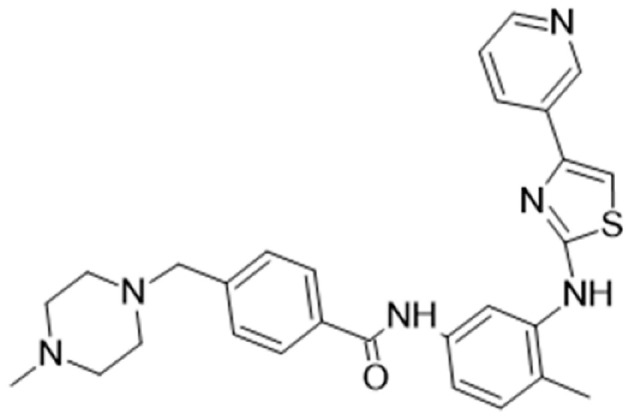	ATP-binding site	Binds to stem cell factor receptor c-KitR,	[137,138,148]
Ceritinib	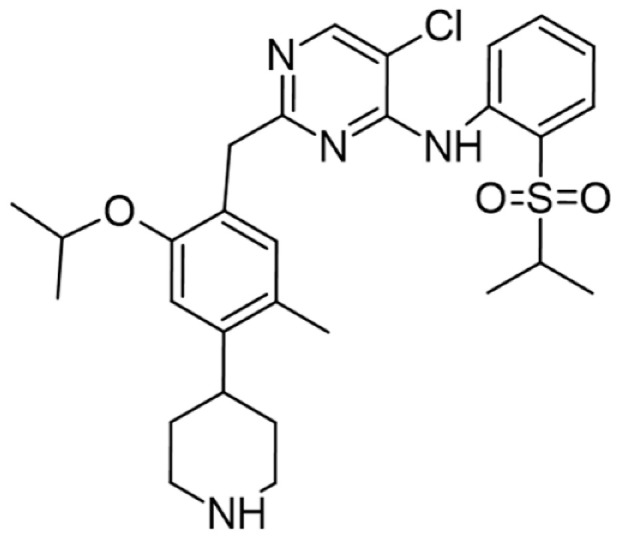	ATP-binding pocket	Autophosphorylation of ALK	[137,138]
Crizotinib	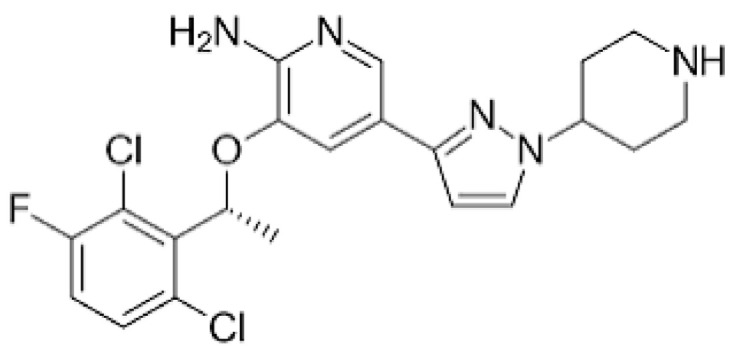	ATP-binding pocket	ALK and c-Met kinase inhibitor	[139,140]
Nilotinib	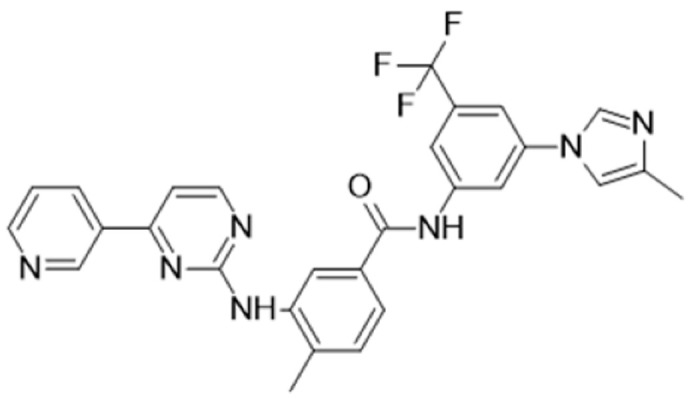	Kinase domain	Chronic myelogenous leukemia	[137,138,139]
A-770041	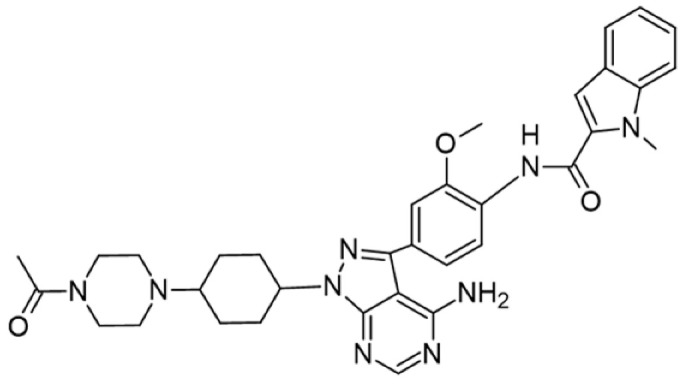	ATP-binding site	Specific Lck inhibitor	[160]
WH-4-23	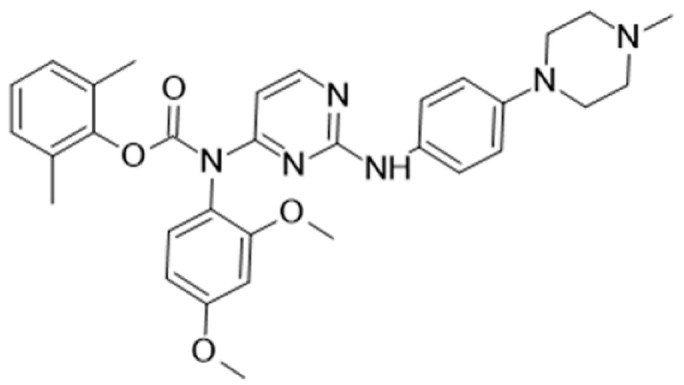	Most probably ATP-binding site	Pan tyrosine kinase inhibitor	[168]
Tirbanibulin	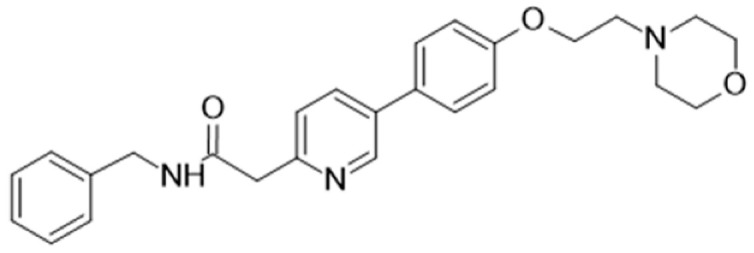	Peptide substrate binding site	Scr inhibtor.	[169]

## Data Availability

No new data were created or analyzed in this study. Data sharing is not applicable to this article.
