# Correction: De Sanctis et al. Lck Function and Modulation: Immune Cytotoxic Response and Tumor Treatment More Than a Simple Event. *Cancers* 2024, *16*, 2630

**DOI:** 10.3390/cancers16234117

**Published:** 2024-12-09

**Authors:** Juan Bautista De Sanctis, Jenny Valentina Garmendia, Hana Duchová, Viktor Valentini, Alex Puskasu, Agáta Kubíčková, Marián Hajdúch

**Affiliations:** 1 Institute of Molecular and Translational Medicine, Faculty of Medicine and Dentistry, Palacky University, 77900 Olomouc, Czech Republic; jenny.garmendia@gmail.com (J.V.G.); viktor.valentini@upol.cz (V.V.); agata.kubickova@upol.cz (A.K.); marian.hajduch@upol.cz (M.H.); 2 Czech Advanced Technologies and Research Institute (CATRIN), 77900 Olomouc, Czech Republic; 3 Faculty of Science, Palacky University, 77900 Olomouc, Czech Republic; hana.duchova01@upol.cz (H.D.); alex.puskas02@gmail.com (A.P.); 4 Laboratory of Experimental Medicine, University Hospital Olomouc, 77900 Olomouc, Czech Republic

## Figure 3 Legend

In the original publication [1], there was a mistake in the legend of Figure 3. The mistake was that we did not reference Borowicz et al., who proposed the model that was modified in this figure in the manuscript.

The correct legend appears below.

**Figure 3.** The figure depicts the role of CD45 and other phosphatases on Lck phosphorylation at residue Y192. This figure was modified from the proposed model described by Borowicz et al. [53]. The phosphorylation can be dependent (susceptible) on CD45 dephosphorylation, or it can be resistant to phosphorylation and maintain the enzyme in close conformation. The thick lines correspond to the preferential function of the enzyme, the physiological response. Other phosphatases can dephosphorylate residues Y192, Y394, and Y505 to generate a nonfunctional enzyme. The previous model differed in its assessment of the importance of Tyr192 for TCR signaling, the hyperphosphorylation of the Tyr192 residue, the role of free Lck, and the role of other phosphatases. This figure was made using Bio Render software.

## Reference

53. Borowicz, P.; Sundvold, V.; Chan, H.; Abrahamsen, G.; Kjelstrup, H.; Nyman, T.A.; Spurkland, A. Tyr192 Regulates Lymphocyte-Specific Tyrosine Kinase Activity in T Cells. *J. Immunol.* **2021**, *207*, 1128–1137. https://doi.org/10.4049/jimmunol.2001105.

With this correction, the order of some references has been adjusted accordingly. The authors apologize for any inconvenience caused and state that the scientific conclusions are unaffected. This correction was approved by the Academic Editor. The original publication has also been updated.
